# Supplementary figures and images for: Intensive pasture management alters the composition and structure of plant-pollinator interactions in Sibiu, Romania
Source: PeerJ. 2024 Feb 29;12:e16900. doi: 10.7717/peerj.16900 (PMC10909354; doi:10.7717/peerj.16900)

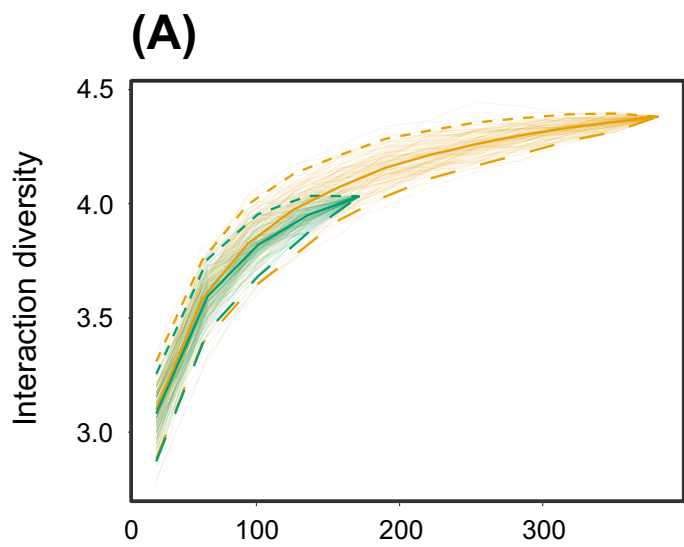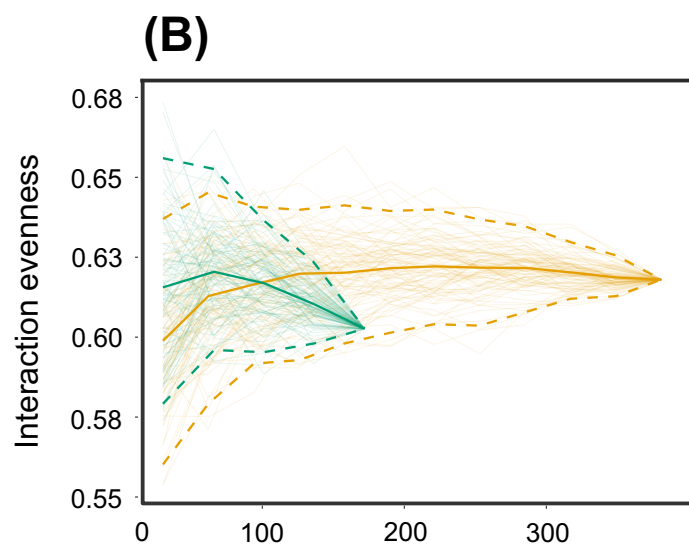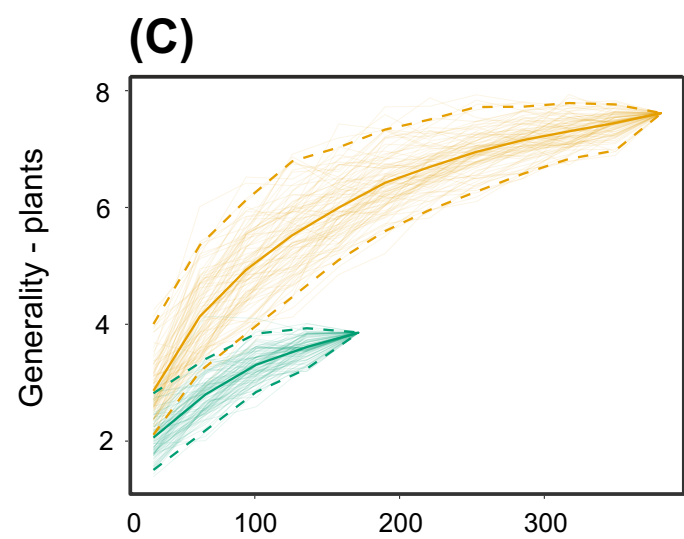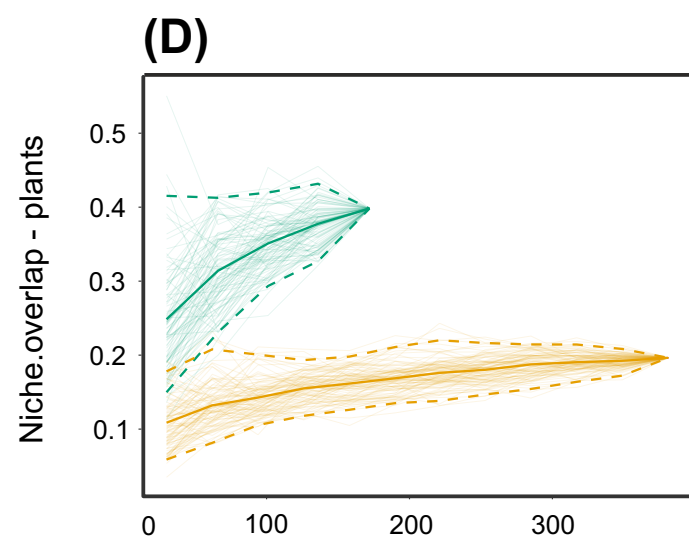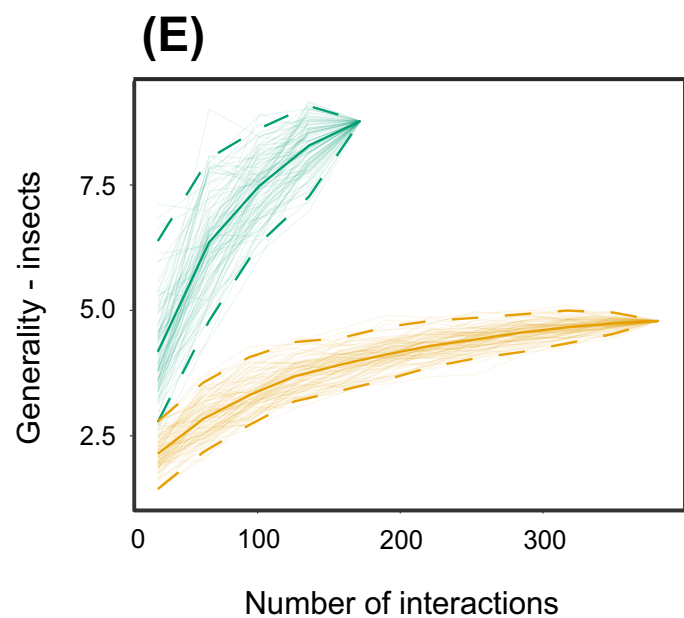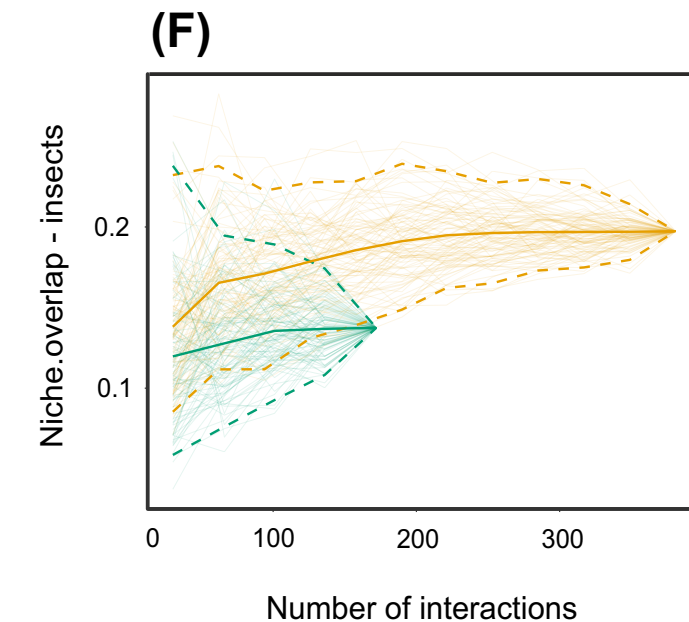

Supplement: Supplemental Information 2 — (A) Shannon diversity of interactions, (B) interaction evenness, (C) generality of plants, (D) niche overlap of plants, (E) generality of pollinators and (F) niche overlap of pollinators are compared between hay meadows (orange) and pastures (blue). Solid lines and dotted lines indicate mean values and 95% confidence intervals of rarefaction estimates based on 100 iterations, respectively. The endpoint of the curve corresponds to the same value generated by the ‘networklevel’ function in the bipartite R package (Dormann et al., 2009) . [file peerj-12-16900-s002.pdf]

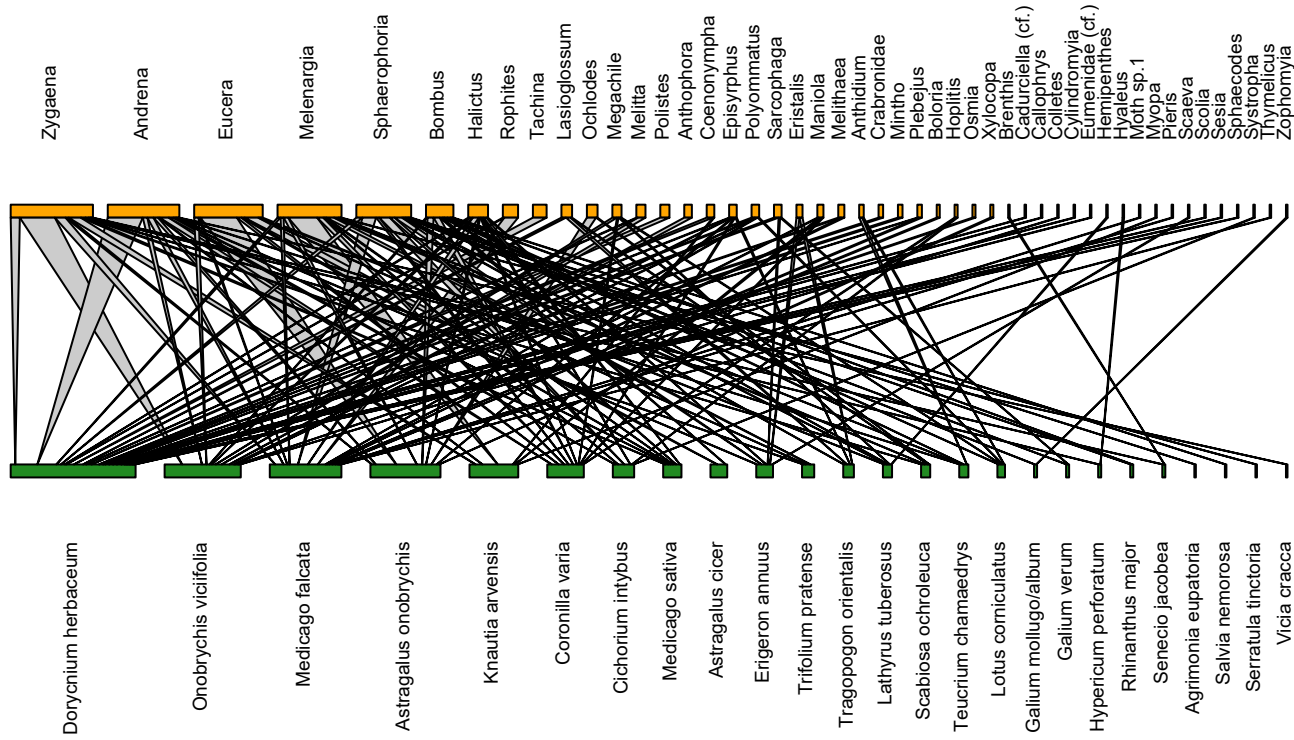

Supplement: Supplemental Information 3 — Plants nodes are shown in green and pollinator nodes in red. The number of interactions are illustrated by line thickness and node size. [file peerj-12-16900-s003.pdf]

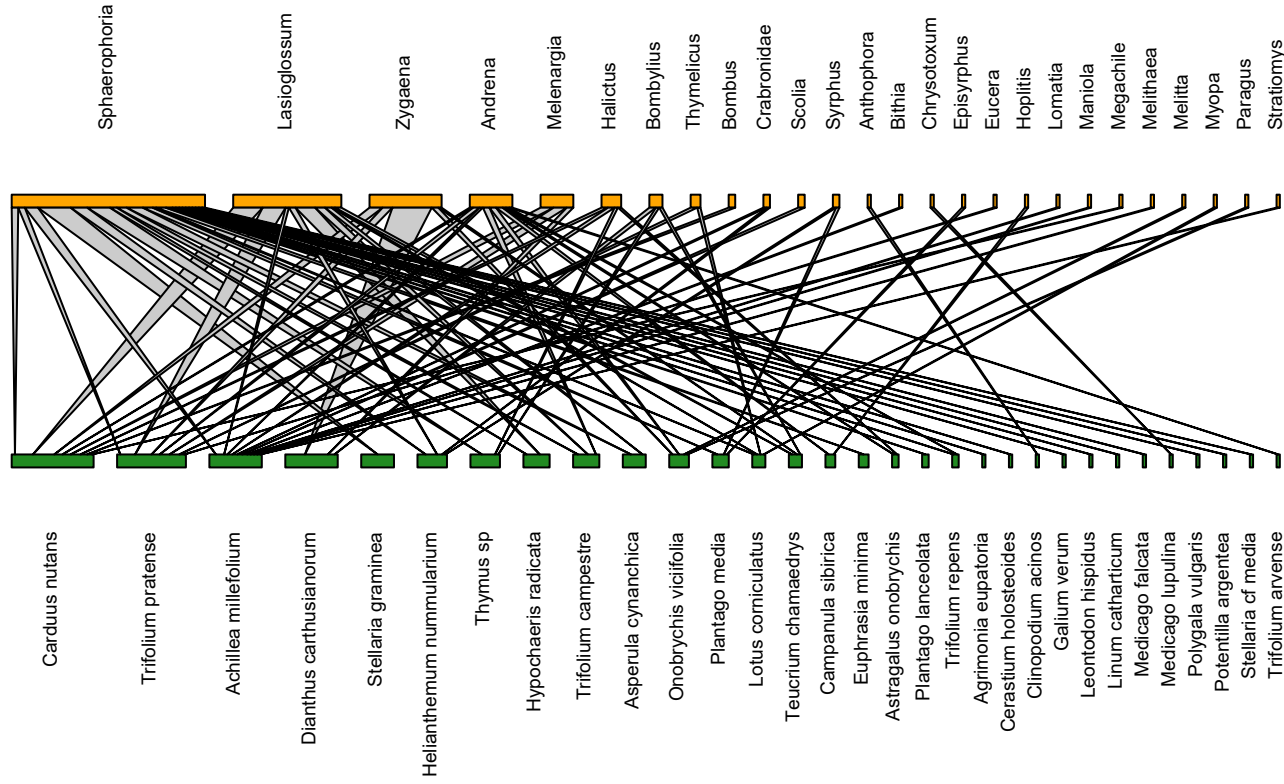

Supplement: Supplemental Information 4 — Plants nodes are shown in green and pollinator nodes in red. The number of interactions are illustrated by line thickness and node size. [file peerj-12-16900-s004.pdf]
